# Editorial: Artificial intelligence in colorectal cancers

**DOI:** 10.3389/fonc.2023.1206311

**Published:** 2023-05-23

**Authors:** Pasquale Cianci, Nicola Tartaglia, Antonio Ambrosi, Enrico Restini

**Affiliations:** ^1^ Department of Surgery and Traumatology, Azienda Sanitaria Locale della Provincia di Barletta Andri Trani (ASL BT), Andria, Italy; ^2^ Department of Medical and Surgical Sciences, University of Foggia, Foggia, Italy

**Keywords:** artificial intelligence, colorectal cancers, robotic surgery, machine learning algorithms, surgery

Colorectal cancer (CRC) is a growing disease in the world, thanks also to the screening programs that have recently been launched in many countries. It represents the third cause of cancer in males, the second in female patients and the second in the world for cancer mortality (1, 2). More than 1.9 million new colorectal cancer cases and 935,000 deaths were expected in 2020 (2). Colorectal cancer is generally considered an indicator of the socio-economic status of a country or population, in fact, in countries previously considered to be at low risk (Eastern Europe, East and Central-Southern Asia and South America) and which today have a phase of great development, increasing incidence rates are observed. Progress has probably produced a change in food and lifestyle. On the other hand, there is a reduction in incidence in these countries once considered high risk (United States, Western Europe), the explanation for this could depend on healthier lifestyle choices and above all on the adoption of mass screening. Colonoscopy performed during screening detects earlier lesions that may be amenable to definitive treatment, but this is showing an increased incidence in patients under 50 years of age. In a recent randomized controlled trial the authors conclude that the 10-year risk of colorectal cancer is lower among participants who undergo screening colonoscopy than among those not assigned to any screening (3). Continuous technological progress is making the application of Artificial Intelligence (AI) increasingly available in our daily lives. Researchers are discovering that thinking systems can have many potential applications in everyday life. Deep learning and machine learning are the branches of AI used to learn data representations with multiple levels of abstraction, which are then processed by algorithms that indicate how much change there should be compared to its previous internal parameters (4). Currently the impact of this technology cannot be quantified, which allows us to imagine how this circumstance could be used for the health needs of the general public, and especially for cancer patients. With these systems there are no fixed limits to their use, but we could also find ourselves faced with unnecessary applications, however the continuous experimentation is slowly delimiting the boundaries. Artificial intelligence has long been used in many fields: medicine, business and relationship life. In medical sciences it is mainly used for the diagnosis, treatment and prognosis of diseases (5, 6). The continuous application of AI in the medical field is increasing the prospects for its use for the diagnosis and treatment of neoplastic diseases. Recently some authors have shown that AI can also play an important role in the diagnosis and treatment of patients with colorectal cancer and can improve the screening efficiency and the 5-year survival rate of these patients after the treatment (7). In this Research Topic, we have involved authors from different countries such as China, Canada, the United States and Romania, in this way we hope to offer a broad point of view on these malignancies. The topics covered concern some of the most important and debated ones on colorectal cancer. The importance of risk factors, early screening, treatments possibility and prognosis has already been stressed, Mansur et al. offer a literature overview of the role of AI in predicting risk, prognosis, and response to therapies among patients with CRC. Their contribution examines various aspects of colorectal cancer and what are the roles that AI is assuming in diagnostics, histopathology, blood testing, imaging, metastasis detection, genetics, therapy and prognosis, not forgetting the future perspectives. Yu et al. performed an extensive review on the role of the microbiome in colorectal cancer. Bioinformatics analysis was performed and a machine learning based Latent Dirichlet Allocation (LDA) model was used for identification of subfield Research Topics. Out of a total of 5696 publications they extrapolated 50 which were divided into four groups of interest, including ‘microbiome sequencing and cancer’, ‘microbiome compositions, interactions and treatment’, ‘microbiome molecular characteristics and mechanisms’ and ‘microbiome and metabolism”. Other important aspects that we wanted to explore concerned colorectal cancer metastases, a robotic approach, outcomes after surgical treatment, the role of AI in predicting the therapeutic response of these ocological patients, and finally, the risk factors in the low anterior resection syndrome (LARS) after surgery for colorectal cancer. This collection does not claim to offer the reader definitive results, but a starting point for future fields of application and research. AI is now a reality that in our opinion needs to be investigated and implemented paying particular attention to taking concrete paths that may actually have future utility such as early screening, genetic research, therapeutic response of these patients to multimodal treatments.

## Author contributions

All authors listed have made a substantial, direct, and intellectual contribution to the work and approved it for publication.
